# Cutaneous squamous cell carcinoma in norway 1963–2011: increasing incidence and stable mortality

**DOI:** 10.1002/cam4.404

**Published:** 2015-01-26

**Authors:** Trude E Robsahm, Per Helsing, Marit B Veierød

**Affiliations:** 1 The Cancer Registry of Norway, Institute of Population-based Cancer ResearchPB 5313 Majorstuen, N-0304, Oslo, Norway; 2 Department of Dermatology, Oslo University Hospital-RikshospitaletN-0027, Oslo, Norway; 3 Department of Biostatistics, Institute of Basic Medical Sciences, Faculty of Medicine, University of OsloPB 1122, N-0317, Oslo, Norway

**Keywords:** Cutaneous squamous cell carcinoma, incidence, mortality, population-based, survival

## Abstract

The incidence of cutaneous squamous cell carcinoma (SCC) is rapidly increasing in white populations, causing high morbidity and health-care costs. Few studies, however, have described the trends for SCC, as population-based data with a long follow-up are limited. In Norway we have this opportunity and we aimed to describe SCC incidence, mortality and survival rates, according to sex, age, stage, primary anatomical location, and geographical region, for the period 1963–2011, for estimation of future health-care needs. Data were retrieved from the Cancer Registry of Norway. Age-adjusted SCC incidence and mortality rates and 5-year relative survival (in percent) were calculated for 5-year calendar periods. A joinpoint regression model identified the annual percentage change (APC) in rates over the 50-year period. The age-adjusted incidence rate increased ninefold in females and sixfold in males from 1963 to 2011, with APCs of 5.6% (95% confidence interval, CI 4.5, 7.3) and 3.3% (95% CI 1.3, 5.3) in females and males, respectively. SCC incidence rose in all age groups, anatomical locations (except ears in females), and geographical regions, though restricted to localized tumors. Most striking increase was seen in the age group 70–79, in face and head locations and among residents in southern Norway. SCC mortality and survival rates remained relatively stable. Our findings underline an increasing need for SCC treatment in Norway, especially considering the aging population. The findings also call for the creation of particular guidelines for primary prevention of SCC.

## Introduction

The incidence of cutaneous squamous cell carcinoma (SCC) is increasing in white populations, worldwide 1. Strong epidemiological evidence links SCC risk to accumulated doses of ultraviolet (UV) radiation, both from the sun and from sunbeds 2–4. Australia and the southern states of the United States have the highest SCC incidence rates, but several countries at higher latitudes, with colder climates and far lower UV doses, such as Northern Europe, have SCC incidence rates that are rapidly increasing 1. However, as many countries lack population-based data on SCC incidence, mortality, and survival, details are sparse 1,5.

From the early 1970–2005, Norway had the highest SCC incidence rate in Scandinavia. After 2005, Danish and Swedish males and Icelandic and Swedish females had the highest SCC incidence rates 6. Between 1978 and 2007, Denmark experienced a two- and threefold increase in SCC incidence in males and females, respectively 7. Data from Sweden showed a fourfold increase in the SCC incidence rate in both sexes from 1960 to 2004 8. In both countries, even stronger increases have occurred in more recent years 6.

Population-based data from Norway have been reported previously, covering the time periods from 1955 to 1988 9 and from 1966 to 1995 10. SCC incidence, mortality, and survival rates after 1995, however, have not yet been studied. In 2011, SCC was the fourth most common cancer among Norwegians aged 70 years or older, representing more than 8% of all cancer cases in this group 11.

Although the prognosis for SCC is good, SCC is considered to be one of the most costly cancers due to its high incidence and expensive treatment needs 12,13. Thus, the monitoring of SCC incidence is of great importance to public health officials, for estimation of future health-care needs and to help guide prevention initiatives. Therefore, we aimed to describe SCC incidence, mortality, and survival rates and trends in Norway, from 1963 to 2011, according to sex, age, stage, primary anatomical location, and geographical region.

## Material and Methods

The Cancer Registry of Norway was established in 1952, at which time reporting of all new cancer cases became mandatory under the law (except for cutaneous basal cell carcinoma). Cases in the Cancer Registry of Norway are identified through a unique 11-digit personal identification number, which is assigned to all newborns and persons residing in Norway, and mandatory reporting from several independent sources ensures completeness and high data quality 14. Due to several changes in the coding of nonmelanoma skin cancers in Norway prior to 1960, and to ensure that only SCCs were included in our data set, the present study includes histologically verified invasive SCCs diagnosed in the Norwegian population from 1963 through 31 December 2011. SCC cases were selected based on the code 191 from the International Classification of Diseases Revision 7 (ICD-7), using the histology codes 8053, 8073, 8074, 8075, 8077, and 8097 (for the time period 1963–1992) and the morphology codes 80703, 80713, 80723, 80733, 80743, 80753, 80763, 80513, 80523, 80943, and 80953 (for the time period 1993–2011).

Information on sex, age at diagnosis, year of diagnosis, metastases, primary anatomical location of the tumor, and geographical region was retrieved from the Cancer Registry of Norway. The follow-up period was divided into 5-year diagnostic time periods (1963–1967, 1968–1972, …, 2008–2011), and age at diagnosis was divided into five age groups (<30, 30–49, 50–69, 70–79, ≥80 years). Based on information on metastases at the time of diagnosis, two stage categories were used: localized (invasive cancer without any metastases) and advanced (any infiltration into surrounding structures, regional or distant metastases). Although minor changes may have occurred over time with regard to coding of SCC metastases, this might not influenced the two stage categories used here.

**Figure 1:**
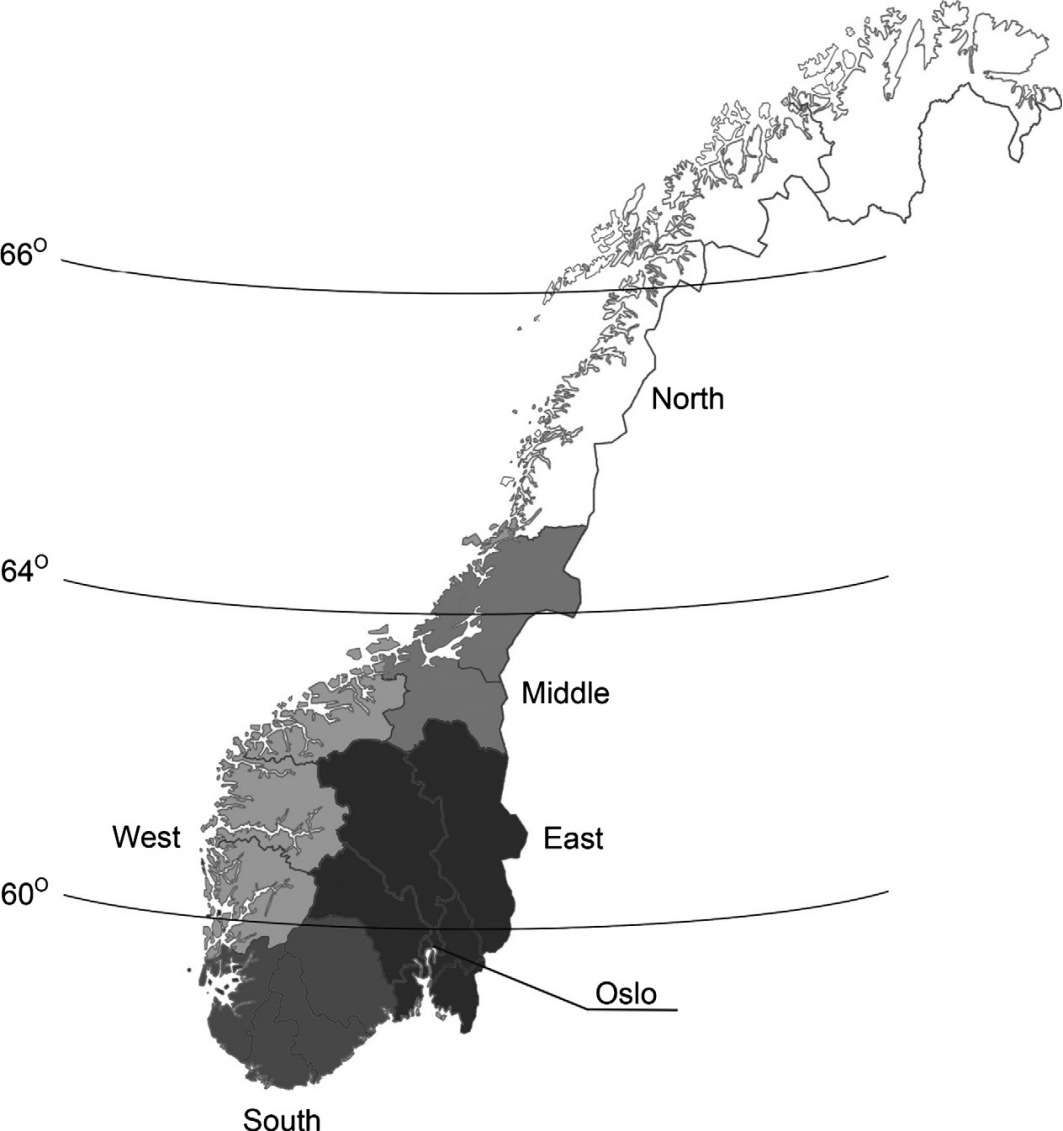
Map of Norway, illustrating the position of the five geographical regions; South, East, West, Middle, and North.

Primary anatomical location of the tumor was categorized using ICD-7 codes: ear (1910), head/face (1911 + 1912), trunk (1913 + 1914), arm (1915), leg (1916), multiple (1918; based on clinical notification of more than one tumor, within the same skin-area and with the same morphology code) and unspecified site (1909). Norway was divided into five geographical regions (South, East, West, Middle, and North) based on the degree of latitude and the UV dose index 15, which is illustrated in Figure1. The Norwegian population is composed principally of Caucasians (>90%). Of the about 5 million inhabitants, 14% are immigrants, of which only a minority is from Asia (4.6%) or Africa (1.8%) 16. However, immigrants from Asia, Africa, and South America comprise ∽20% of the inhabitants of the capital city Oslo 16, thus separate analyses were performed for Oslo.

### Statistics

The incidence and mortality rates were calculated using the computer packages *Incidence* and *Mortality*, which give access to the database of the Cancer Registry. The age-adjusted SCC incidence and mortality rates were standardized based on direct standardization according to the European Standard Population and rates were calculated according to sex, age and stage. Incidence rates were also calculated by anatomical location and geographical region. The rates were expressed per 100,000 person-years. The survival analysis was conducted with a cohort approach using the Pohar Perme method 17, which is implemented in STATA 18. The Pohar Perme estimator calculates 5-year net survival, which is percent alive after 5 years, given that SCC is the only cause of death, using the mortality rate in the Norwegian population as background. All data on SCC cases and deaths were obtained from the Cancer Registry database.

A joinpoint regression model was used to identify changes in the age-adjusted SCC incidence rates over the 50-year time period 19. We fitted the simplest model specifying zero joinpoints (a straight line). The annual percentage change (APC) for the period 1963–2011 was estimated by sex, age, stage, anatomical location, and geographical region.

Predictions of future SCC incidence rates and absolute numbers of cases were conducted using the Nordpred package for cancer projections in NORDCAN (http://www-dep.iarc.fr/NORDCAN/NO/frame.asp), which is an R-package that predicts trends in cancer incidence using an age-period-cohort model 20.

## Results

### Trends in SCC incidence

Throughout follow-up, a total of 30 818 SCC cases were registered. The age-adjusted incidence rates increased ninefold in females and sixfold in males from 1963 to 2011 and the rate was higher in males than in females during the entire study period (Table1). However, the age-specific rate for the age group 30–49 years was higher in females than in males after about 2000. The increase in the SCC incidence rate was almost linear over time in both sexes. APC was 5.6% (95%, CI 4.5, 7.3) in females and 3.3% (95% CI 1.3, 5.3) in males. An increase in SCC incidence rate was seen in all age groups over 30 years of age, but the most striking increase was observed in the age group 70–79 years.

**Table 1:** Incidence rates of cutaneous squamous cell carcinoma, number of cases (*n*) and annual percentage change (APC) with 95% confidence interval (CI) for females and males, by age and stage of disease at diagnosis, stratified by calendar period, 1963–2011

	5-year calendar period										1963–2011		
	1963–1967	1968–1972	1973–1977	1978–1982	1983–1987	1988–1992	1993–1997	1998–2002	2003–2007	2008–2011	*n*	APC	95% CI
Female^1^	1.7	2.4	4.3	4.9	6.0	8.2	10.2	11.1	13.9	15.2		5.6	4.5, 7.3
*n*	177	293	565	737	997	1431	1875	2151	2851	2670	13,747		
Age (years)													
<30	0	0	0	0	0.1	0.1	0	0.1	0.1	0.1	21	–	–
30–49	0.5	0.6	1.0	0.9	1.1	1.6	1.7	2.3	2.5	2.4	418	3.5	1.6, 5.4
50–69	2.7	3.4	6.1	7.3	9.7	14.2	18.4	17.2	21.1	22.0	2730	4.3	3.1, 5.6
70–79	8.2	15.6	25.1	27.5	34.7	49.8	59.2	70.6	87.6	103.9	3907	5.3	4.8, 5.9
≥80	27.1	37.1	66.7	83.2	96.5	117.8	148.2	160.6	215.6	255.1	6671	4.9	4.5, 5.3
Stage^1^													
Localized	1.6	2.2	4.1	4.7	5.8	7.9	10.1	10.9	13.7	13.9	13,461	4.9	3.8, 5.9
Advanced	0.1	0.2	0.1	0.1	0.2	0.3	0.2	0.3	0.2	0.1	286	0.6	−0.4, 1.6
Male^1^	4.0	6.0	8.8	10.0	11.9	14.4	18.4	20.1	21.9	23.7	17,071	3.3	1.3, 5.3
*n*	346	551	884	1084	1375	1762	2309	2633	3136	2991			
Age (years)													
<30	0	0	0	0	0	0	0.1	0.1	0.1	0.1	17	–	–
30–49	0.8	0.7	0.8	1.0	1.3	1.4	1.8	2.3	2.1	1.7	410	1.9	0.2, 3.5
50–69	4.9	8.8	12.6	16.1	19.1	23.3	28.5	27.9	26.8	29.3	4281	4.4	3.4, 5.4
70–79	24.3	34.3	56.4	62.2	79.6	100.4	126.5	140.5	162.5	167.8	6022	4.5	4.1, 4.9
≥80	74.6	101.2	143.8	154.8	178.3	216.4	262.1	290.0	359.0	441.8	6341	3.8	3.5, 4.1
Stage^1^													
Localized	3.9	5.6	8.5	9.8	11.6	13.9	18.1	19.8	21.7	20.2	16,716	4.2	2.4, 6.0
Advanced	0.2	0.4	0.3	0.2	0.3	0.5	0.3	0.3	0.2	0.4	355	0.6	−0.8, 2.0

1 Age-standardized rate, according to the European Standard Population.

Most SCCs were diagnosed as localized, and these are the tumors responsible for the steep increase in SCC incidence (Table1). For advanced tumors, no significant increase was observed, with APCs of 0.6% in both sexes (95% CIs were −0.4, 1.6 in females and −0.8, 2.0 in males).

Most SCC occurred in the head/face area, which was the anatomical location with the steepest increase in incidence rate (Table2). The APCs were 6.1 (95% CI 4.5, 7.8) in females and 5.1 (95% CI 3.2, 6.9) in males. However, a significant increase in incidence rates was seen for all anatomical locations, except for the ear in females, for which the rate was stable. In males, the incidence rate of ear SCC increased significantly, though it plateaued in the 1990s. Starting in the late 1980s, a steep increase in trunk SCC was observed in both sexes. After about 1990, the trunk became the second most common anatomical location in females. In males, the incidence rate of trunk SCC recently exceeded that of ear SCC. Throughout the study period, the APC for trunk SCC was 3.9 (95% CI 2.5, 5.4) and 4.9 (95% CI 3.7, 6.1) in females and males, respectively. When stratifying by age, we observed that females aged 50–69 years and ≥70 years had similar APCs for trunk SCC (6.8%, 95% CI 5.4, 8.3 vs. 6.6%, 95% CI 5.1, 8.2), while in the same age groups in males, a significantly higher APC was found in the oldest age group (5.4%, 95% CI 4.1, 6.8 vs. 8.6%, 95% CI 7.2, 9.9). Over time, a slightly higher incidence rate of arm SCC was seen in males. After about 2000, females had a higher rate of leg SCC. For both arm and leg SCC, slightly higher APCs were observed in females than in males (not significantly different). When the analyses were stratified by age, a steep increase in the incidence rate of arm and leg SCC was found in females ≥50 years of age (data not shown), but in males an increase was only seen in age groups ≥70 years. A steep increase in the incidence rate of multiple SCC was also seen, with APCs of 5.5% (95% CI 3.5, 6.7) and 5.4% (95% CI 2.9, 7.7) in females and males, respectively (Table2).

**Table 2:** Incidence rates^1^ of cutaneous squamous cell carcinoma and annual percentage change (APC) with 95% confidence interval (CI) by sex and anatomical location, stratified by calendar period, 1963–2011.

	5-year calendar period										1963–2011	
	1963–67	1968–72	1973–77	1978–82	1983–87	1988–92	1993–97	1998–02	2003–07	2008–11	APC	95% CI
Female												
Ear	0.2	0.2	0.2	0.1	0.2	0.2	0.2	0.2	0.2	0.2	0.6	−0.2, 1.4
Head/face	0.8	1.2	2.6	3.2	4.0	4.7	6.0	6.2	6.8	7.3	6.1	4.5, 7.8
Trunk	0.3	0.4	0.5	0.6	0.7	1.3	1.6	1.8	2.7	3.4	3.9	2.5, 5.4
Arm	0.3	0.2	0.4	0.5	0.7	0.9	1.1	1.2	1.4	1.3	4.7	3.6, 5.9
Leg	0.2	0.2	0.3	0.3	0.3	0.7	0.8	0.9	1.5	1.5	4.3	3.1, 5.6
Multiple	0.0	0.1	0.2	0.1	0	0.2	0.5	0.5	1.0	1.1	5.5	3.5, 6.7
Unspecified	0	0	0.1	0.1	0.1	0.2	0.2	0.2	0.2	0.4	2.0	1.2, 2.7
Male												
Ear	1.4	1.6	2.1	2.7	3.1	3.3	3.3	3.3	3.7	3.9	2.0	0.4, 3.5
Head/face	1.6	2.6	3.8	4.5	5.5	6.9	8.6	9.7	9.6	10.4	5.1	3.2, 6.9
Trunk	0.3	0.5	0.8	0.9	1.1	1.7	2.9	3.3	3.7	4.3	4.9	3.7, 6.1
Arm	0.5	0.7	1.0	1.1	1.3	1.4	1.6	1.6	1.6	1.5	3.5	2.3, 4.7
Leg	0.2	0.2	0.3	0.3	0.5	0.5	0.6	0.7	0.9	0.8	3.0	1.8, 4,2
Multiple	0.1	0.4	0.6	0.4	0.3	0.5	1.0	1.2	2.1	2.5	5.4	2.9, 7.7
Unspecified	0.1	0	0.1	0.1	0.1	0.2	0.3	0.3	0.3	0.5	3.3	2.7, 4.0

1 Age standardized rates, according to the European Standard Population.

**Figure 2:**
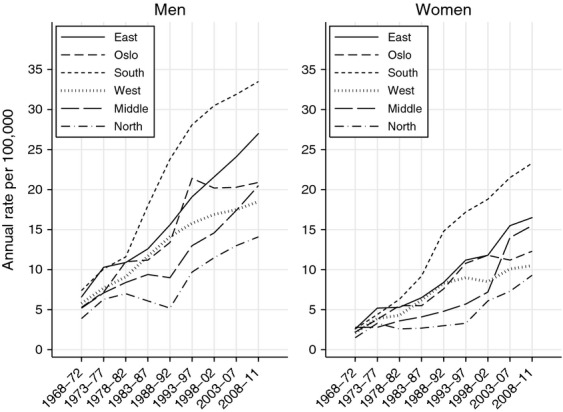
Age-adjusted incidence rates of cutaneous squamous cell carcinoma by geographical region and sex in Norway, 1963–2011.

SCC incidence rates in the South of Norway were twice those in the North throughout the study period (Fig.2). In both sexes, the steepest rate increase was seen in the South (APCs were 6.7 (95% CI 5.4–8.1) in females and 6.3 (95% CI 4.3–8.4) in males). In males, however, a similar increase was seen in the West (APC 6.1 [95% CI 4.7, 7.5]). When stratifying by anatomical location we found that this was due to a steep increase in ear and trunk SCC (data not shown). In females, residents in the Middle and North regions also experienced a strong increase in incidence rates (APCs were 5.4 [95% CI 3.9–6.9] and 4.9 [95% CI 3.2–6.7], respectively). After 2000, the rate for females in the Middle region was close to that of the East region. This pattern was observed for all anatomical locations (data not shown). In Oslo, SCC incidence rates rose significantly until the early 1990s, when the rate leveled off in both sexes, until they were below the rate in the surrounding East region (Fig.2).

Predictions of the future showed that the increase in age-adjusted incidence rates are not expected to continue after 2020, but the annual number of new SCC cases is expected to increase (data not shown). In the 5-year period 2007–2011, the average annual numbers of new cases were 680 in females and 760 in males. The corresponding numbers are expected to be 1041 and 1222, respectively, in the 5-year period 2027–2031.

### Mortality trends

During the 50-year study period the mortality rate for males remained unchanged (APC −1.2, 95% CI −2.5, 0.2) while a slight decrease was observed for females (APC −2.0, 95% CI −2.9, −1.1). Analyses by age revealed that this decrease was restricted to the oldest age groups (≥70 years). No differences were observed between geographical regions, neither in males nor in females (data not shown). Due to small numbers, separate analyses by anatomical location were not conducted.

### Survival trends

Table3 presents the 5-year relative survival from 1963 to 2011 by age, stage, and anatomical location. SCC survival among females improved slightly over time, while this was not found in males (Table3). The improvement in female survival was most pronounced after ear, trunk and arm/leg SCC. Five-year relative survival after localized SCC was high and changed less than 5-year relative survival after advanced SCC in both sexes. Here, an improvement was seen, though it was most pronounced in females. No differences in survival were observed between geographical regions (data not shown).

**Table 3:** Five-year relative survival rates (SR) with 95% confidence intervals (CIs), for cutaneous squamous cell carcinoma in females and males, by age, anatomical location, and stage of disease

	SR (95% CI)				
	1963–1969	1970–1979	1980–1989	1990–1999	2000–2011
Female					
Age (years)					
<30	1.00	1.00	1.00	1.00	1.00
30–49	0.76 (0.46–0.90)	0.85 (0.69–0.92)	0.89 (0.76–0.95)	0.89 (0.81–0.93)	0.98 (0.93–0.99)
50–69	0.75 (0.62–0.83)	0.89 (0.82–9.92)	0.91 (0.86–0.94)	0.94 (0.91–0.95)	0.95 (0.92–0.96)
70–79	0.70 (0.52–0.82)	0.88 (0.80–0.93)	0.96 (0.89–0.98)	0.90 (0.87–0.93)	0.94 (0.91–0.96)
≥80	0.76 (0.41–0.92)	0.87 (0.67–0.95)	0.85 (0.75–0.92)	0.89 (0.83–0.93)	0.82 (0.77–0.86)
Location					
Face/head	0.94 (0.32–0.99)	0.93 (0.81–0.97)	0.90 (0.84–0.94)	0.93 (0.88–0.96)	0.86 (0.82–0.89)
Ear	0.33 (0.12–0.55)	0.61 (0.37–0.78)	0.68 (0.43–0.83)	0.77 (0.50–0.90)	0.92 (0.42–0.99)
Trunk	0.60 (0.40–0.75)	0.83 (0.63–0.93)	0.87 (0.71–0.94)	0.88 (0.79–0.93)	0.86 (0.80–0.90)
Arm	0.58 (0.31–0.77)	0.66 (0.47–0.79)	0.96 (0.45–0.99)	0.88 (0.77–0.94)	0.89 (0.78–0.94)
Leg	0.69 (0.37–0.87)	0.89 (0.47–0.98)	0.89 (0.65–0.97)	0.88 (0.76–0.94)	0.91 (0.81–0.95)
Multiple	0.86 (0.00–0.99)	0.98 (0.00–1.00)	0.91 (0.36–0.99)	0.81 (0.70–0.89)	0.91 (0.81–0.95)
Stage					
Localized	0.79 (0.66–0.88)	0.91 (0.83–0.95)	0.91 (0.86–0.94)	0.91 (0.88–0.93)	0.88 (0.85–0.90)
Advanced	0.17 (0.05–0.36)	0.21 (0.09–0.37)	0.43 (0.25–0.58)	0.74 (0.54–0.87)	0.64 (0.45–0.78)
Male					
Age (years)					
<30	1.00	1.00	1.00	0.80 (0.20–0.97)	0.80 (0.20–0.97)
30–49	0.85 (0.61–0.95)	0.90 (0.74–0.96)	0.89 (0.77–0.94)	0.90 (0.81–0.94)	0.92 (0.86–0.95)
50–69	0.90 (0.79–0.95)	0.90 (0.85–0.93)	0.92 (0.88–0.94)	0.90 (0.87–0.92)	0.89 (0.87–0.90)
70–79	0.84 (0.68–0.92)	0.89 (0.81–0.83)	0.88 (0.83–0.92)	0.87 (0.84–0.90)	0.85 (0.83–0.88)
≥80	0.80 (0.52–0.92)	0.79 (0.65–0.87)	0.84 (0.73–0.91)	0.79 (0.72–0.84)	0.75 (0.71–0.79)
Location					
Face/head	0.81 (0.65–0.90)	0.88 (0.79–0.94)	0.87 (0.80–0.91)	0.86 (0.81–0.89)	0.79 (0.76–0.82)
Ear	0.87 (0.63–0.96)	0.87 (0.75–0.93)	0.92 (0.80–0.97)	0.84 (0.75–0.89)	0.83 (0.77–0.88)
Trunk	0.74 (0.48–0.88)	0.69 (0.56–0.79)	0.88 (0.74–0.95)	0.90 (0.82–0.94)	0.85 (0.79–0.89)
Arm	0.76 (0.39–0.92)	0.83 (0.67–0.92)	0.82 (0.69–0.89)	0.88 (0.76–0.94)	0.81 (0.71–0.87)
Leg	0.90 (0.05–0.99)	0.62 (0.32–0.81)	0.98 (0.00–1.00)	0.94 (0.58–0.99)	0.91 (0.76–0.97)
Multiple	1.00	1.00	0.85 (0.07–0.99)	0.78 (0.70–0.84)	0.83 (0.76–0.87)
Stage					
Localized	0.88 (0.77–0.94)	0.88 (0.83–0.92)	0.89 (0.85–0.91)	0.86 (0.83–0.88)	0.82 (0.80–0.84)
Advanced	0.25 (0.08–0.45)	0.21 (0.09–0.36)	0.56 (0.38–0.71)	0.71 (0.49–0.85)	0.51 (0.34–0.64)

## Discussion

We found a strong increase in SCC incidence rates for both females and males over time. The rates increased significantly in all age groups ≥30 years, at all anatomical locations (except ears in females) and geographical regions, although the most striking increases were seen in the age group 70–79 years, in face/head locations and in the South of Norway. However, this increase in incidence was restricted to localized tumors.

UV exposure is considered the main environmental cause of SCC, and SCC is the skin cancer type that has the most straightforward relation to accumulated UV dose 3,21. Since early 1990, the Norwegian Cancer Society, a nationwide, nonprofit voluntary organization, has run sun awareness campaigns and communicated public guidelines to prevent skin cancer. In spite of this, our results indicate increasing UV exposure in the Norwegian population. In a recent survey conducted by the Norwegian Cancer Society, participants reported to have knowledge about sun protection strategies, but also to have sun-bathing habits that constitute known risk factors for skin cancer 22. Furthermore, about 30% of the participants reported annual holidays abroad at sunny destinations, and more than 50% reported sunburns during the last 12 months, although an increasing use of sunscreen was observed. Frequent sunburn among sunscreen users has been reported in observational studies, and this paradox may be due to inappropriate use of sunscreens, which then give less protection than expected 23. The campaigns and guidelines, however, have primarily advocated avoidance of sunburns by intermittent UV exposure as cutaneous malignant melanoma (CMM) prevention. Moreover, increased public awareness may also increase diagnostic intensity, and thus the raise the SCC incidence. Another factor that may have contributed to the higher SCC rates is the increasing number of organ transplantations. There is a strong association between long-term use of immune-suppressive drugs and the risk of SCC 24. In Norway, the first organ transplantation was conducted in 1956 25. Thereafter, the number of transplantations has steadily risen; today about 450 transplantations are conducted annually 26.

Throughout the last 50 years, males had the highest SCC incidence rate, while females tended to have a steeper rate increase. Since the age-specific incidence rates increased, the aging of the population alone cannot explain these elevated rates. In particular, the increase in females aged 50–69 years during the last 10–15 years is noteworthy. This pattern has also been observed in Denmark 7 and Sweden 8,27. UV radiation from sunbeds increases the risk of SCC 4. As sunbeds became popular in Norway during the 1980s, and younger females in particular have been reported to be common users 22, one may speculate that sunbeds contributed to the elevated SCC incidence rates in females aged 50–69 years.

The most common anatomic location was the face/head, which also had the steepest increase in incidence rate, in agreement with previously reported results from Europe 5,28, and Australia 29. The face and head are often exposed to UV radiation and our findings support the role of accumulated UV exposure in SCC. However, only a modest increase was seen for ear SCC among males, although the UV dose should be similar for the ears and the face/head. This pattern has also been observed in Sweden, where less use of headgear (e.g., hats) in males was suggested as a possible explanation 8. As headgear does not usually cover the ears, less use will not impact the UV dose received by the ears, but will significantly increase the UV dose received by areas such as the scalp and forehead. The lower risk of ear SCC in females may result from more protection offered by their hair 10.

The increase in trunk SCCs was strong during the study period, and for the most recent time period, the trunk was the second most common anatomical location in both sexes. The trunk is also the most common location for CMM in Norway, with the steepest increase in incidence rate over time 30. The suggested explanation for this includes changes in fashion trends, which included exposing the trunk, and sun exposure habits, in particular in the elderly (>60 years). Although the etiology of SCC differs from that of CMM, it is reasonable to believe that the increase in trunk SSCs can be attributed to the same reasons. The multiple SCC incidence rate also rose significantly in both sexes. However, the most striking increase was observed in the oldest age group and in the sunniest geographical regions (the South and East). It is thus reasonable to believe that a change in sun exposure habits in the elderly has elevated the accumulated UV dose over time.

The increase in leg SCC was most pronounced in females. Norwegian females also have a high incidence of leg CMM 30, but contrary to the pattern observed for leg SCC, the rate of leg CMM has decreased in Norwegian females in the last 10–15 years. In general, SCCs located at the extremities increased more steeply in females than in males after the age of 50.

Throughout follow-up, the SCC incidence rate in the South was twice that in the North. Long-term UV data have been reconstructed for Norway, and area of residence was shown to reflect everyday UV exposure, as it fit well with the geographical variation in CMM risk 31. However, we also observed a strong increase in SCC incidence rates in the West (males), Middle, and North (females) regions, which have far less sunny days and a colder climate compared to the South 32. This may result from increased travel and holidays to sunny regions abroad. In Oslo, a modest increase in SCC incidence rate was found, compared to the East region (located at the same latitude, and with the same climate). The relatively high proportion of non-Western immigrants residing in Oslo might explain this difference, and when calculating the incidence rate after excluding parts of the city with a high density of immigrants, the rate became similar to the rate in the East (data not shown).

Localized tumors were found to account for all of the increases in SCC incidence rates, which explain the stable and relatively high survival rates observed in all age groups in Norway. However, when anatomical location was considered, significant improvements in survival were seen in females for SCCs of the ear, trunk, and extremities. Norwegian females were previously reported to have lower SCC survival than males for tumors of the same anatomical location, which was suggested to result from patient delay 10. However, these sex differences do not seem to apply today.

With regard to the future, we do not expect the increase in age-adjusted SCC incidence to continue, we nevertheless expect an annual increase in new SCC diagnoses over the next 20 years, due to population aging.

The major strength of this study is the high-quality data and the long-term follow-up. Most SCCs in the Cancer Registry of Norway were morphologically verified (99.8% in the period 2001–2005) 14. Nevertheless, we cannot exclude the possibility that some cases were missed, as SCC has a low lethal potential and not all cases are necessarily diagnosed or treated, in particular in elderly people with comorbidities.

In conclusion, we observed significantly increased SCC incidence rates in both sexes. The increase in females is especially noteworthy. A strong incidence increase in less sunny regions indicates that sun-holidays abroad elevate the risk of SCC. Most of the SCC was observed to occur in the face/head, which will cause increasing cosmetic and functional morbidity as time goes on. Our findings underline a need of guidelines for the prevention of SCC and an increasing need for SCC treatment, especially when considering the aging of the population.

## Conflict of Interest

None declared.
